# Evolutionary potential and adaptation of *Banksia attenuata* (Proteaceae) to climate and fire regime in southwestern Australia, a global biodiversity hotspot

**DOI:** 10.1038/srep26315

**Published:** 2016-05-23

**Authors:** Tianhua He, Haylee D’Agui, Sim Lin Lim, Neal J. Enright, Yiqi Luo

**Affiliations:** 1 Department of Environment and Agriculture, Curtin University, Perth, WA 6845, Australia; 2 School of Veterinary and Life Sciences, Murdoch University, Perth, WA 6150, Australia; 3 Department of Microbiology and Plant Biology, University of Oklahoma, Norman, OK 73019, USA

## Abstract

Substantial climate changes are evident across Australia, with declining rainfall and rising temperature in conjunction with frequent fires. Considerable species loss and range contractions have been predicted; however, our understanding of how genetic variation may promote adaptation in response to climate change remains uncertain. Here we characterized candidate genes associated with rainfall gradients, temperatures, and fire intervals through environmental association analysis. We found that overall population adaptive genetic variation was significantly affected by shortened fire intervals, whereas declining rainfall and rising temperature did not have a detectable influence. Candidate SNPs associated with rainfall and high temperature were diverse, whereas SNPs associated with specific fire intervals were mainly fixed in one allele. Gene annotation further revealed four genes with functions in stress tolerance, the regulation of stomatal opening and closure, energy use, and morphogenesis with adaptation to climate and fire intervals. *B. attenuata* may tolerate further changes in rainfall and temperature through evolutionary adaptations based on their adaptive genetic variation. However, the capacity to survive future climate change may be compromised by changes in the fire regime.

Mediterranean type ecosystems (MTE’s) are among the most biologically diverse terrestrial ecosystems globally and are thought to be highly vulnerable to species loss under global change^1^,^2^,^3^. Both drought and fire play an important role in shaping the structure and composition of MTE vegetation, as the distribution and abundance of plant species is determined primarily by their ability to tolerate water stress and extreme temperatures in the summer, and to re-establish themselves after disturbance, usually from fire. Significant climate trends of warming and drying are already evident across the world’s MTE’s, raising concerns about the consequences for their diverse floras^4^. Moreover, climate change is redefining management strategies and conservation goals and concepts^5^.

Malcolm *et al*. identify South-Western Australia (SWA) and the Cape Region of South Africa as two of the most vulnerable MTE regions globally, potentially losing more than 2000 plant species each over the next 100 years in the face of climate change^6^. The climate of SWA has undergone dramatic change since the mid-1970s, with annual rainfall decreasing by 30% and mean maximum temperature increasing by 0.15–0.20 °C per decade^7^. Global climate models project a further temperature increase of 1–3 °C across all seasons of the year, a further 10–20% reduction in rainfall (largely in winter), and a higher frequency of extreme events such as droughts. In a bioclimatic envelope modeling analysis, Fitzpatrick *et al*. estimated that up to 25% of *Banksia* species (Proteaceae) were projected to become extinct by 2080^8^. Similarly, Yates *et al*. simulated the impacts of climate-change scenarios on *Banksia* species distributions and reported an increased risk of decline for all species^9^. More recently, Urban has predicted that 14% of native species in Australia and New Zealand will become extinct by 2100 if the current trend of climate change continues^10^.

However, the validity of these sobering extinction predictions is uncertain as critical gaps remain in our knowledge of the intrinsic capacity of species to respond to climate and other environmental changes. For instance, species may be able to adapt *in situ* to new climatic conditions based on genetic variation within populations and plasticity, e.g., most species can persist outside their natural range^11^, albeit under altered competition contexts. Recently, attempts to predict the impacts of climate change on biodiversity have moved beyond species-level models and toward a greater consideration of intraspecific variations in tolerances and adaptation^12^,^13^. Broad niche breadth and higher adaptive genetic variation could buffer genotypes from the immediate effects of climate and environmental change^14^. To effectively assess the responses of a species to climate change, we need to understand both the current levels of adaptation within a species and its future adaptive potential^15^. We therefore need to know the level of adaptive genetic variation in extant populations and the variation associated with adaptation to components of climate and other environmental conditions.

SWA is also one of the most fire-prone regions in the world^16^, and plants here display remarkable adaptations to recurrent fire^17^. Although the expected interaction with climate change is complex, a projected hotter and drier climate with more high fire danger days will likely lead to more fire, and so, shorter fire intervals^18^. However, other human activities also play an important role in determining fire regimes. Both historical accounts and evidence from current land-use practices support the argument that Aboriginal peoples used fire as a land management tool over the past 50 k years before the European settlement of Australia, with increased occurrence of fire under their land management in areas with high resource availability^19^. Since the 1950s, the managed use of fire to reduce fuel loads in public estate vegetation types has been the major strategy employed by government agencies in Australia to mitigate the risk of fire spreading into private lands^20^,^21^. Such altered fire frequency (shortened fire intervals) is an important component of environmental change and has been implicated in shifts in community structure^22^, species loss and invasions. Experimental studies show mixed results, with overall species richness adversely affected in shrublands^23^ but not in wetter forests burned at 3–5-year intervals^24^, whereas the abundance of specific plant functional types (e.g., obligate seeding shrubs) was significantly reduced in both.

Plants may respond to climate and environmental changes either by persisting *in situ* through tolerating and/or adapting to the changes, or by migrating to suitable habitats if possible. Recent research has shown that many Australian plant species have the capacity to disperse their seeds over long distances, especially after fire^25^,^26^,^27^,^28^. However, in at least the past 700 k years^29^, and possibly since the mid-Pliocene^30^, most Australian species seem to have persisted through major climatic changes in localized habitats rather than by moving long distances. Species may be able to retreat to nearby refugia in the face of climatic and other types of environmental change, thereby allowing them to persist locally^31^,^32^. This pattern emphasizes the importance of maintaining an adaptive life-history trait set with adequate genetic variation in populations so that species can persist through changing conditions^33^.

Investigating adaptive genetic variation may reveal the role of genetic diversity in buffering species and communities against the effects of changing climate^34^,^35^. Variations in neutral genetic markers (e.g., microsatellite DNAs) have traditionally been used as indicators of the evolutionary potential of wild populations. However, recent studies have questioned the usefulness of molecular indices of neutral genetic variability as surrogates of the evolutionary potential of natural populations, as these markers are generally not under selection^35^,^36^, though such a view overlooks that presently neutral variation may become adaptive if new selection pressures emerge^37^. Nevertheless, the challenge now is to identify whether species harbor sufficient adaptive genetic capacity^12^,^38^. In plants, the functional traits linked to phenology, growth and stress resistance are shaped by selection along environmental gradients (in space and time). Those functional traits exhibiting sufficient genetic variation are expected to facilitate rapid evolutionary adaptation to climate change^39^. Given rapid climate change, the immediate adaptation of populations to recruitment and growing conditions must rely on this existing genetic variation, as these variations were selected over many generations and are capable of providing immediate adaptive value to the population when facing rapid environmental changes^37^. Research is now emerging that takes genetic adaptation and evolutionary capacity into account in predictions of species or ecosystem responses to climate change^39^,^40^,^41^.

The rapidly falling costs of next-generation sequencing are now enabling the genome-wide characterization of adaptive genetic variation, which offers unprecedented power to identify the loci that mediate local adaptation^42^. Recent research has demonstrated the value of single-nucleotide polymorphisms (SNPs) in detecting selection- and adaptation-related candidate genes^40^,^43^. SNPs have clear advantages for accommodating models of evolutionary change and their potential roles in functional evolution. By screening large numbers of SNPs, genome-scale studies open the possibility of identifying loci that mediate fitness in different environments and contribute to local adaptation^44^. Restriction-site-associated DNA sequencing (RAD-seq) combines enzymatic fragmentation of the genome with high-throughput sequencing to generate large numbers of SNP markers^45^. This process enables large-scale studies of genomic variation in species lacking a reference^46^.

*Banksia attenuata* (Proteaceae) is one of the most prominent and widespread woody plants in SWA. It occurs in semi-arid shrubland to mesic forest and is highly resilient to fire, recovering by resprouting from its trunk or base. Here, we utilize RAD-seq to screen large numbers of SNPs and characterize adaptive genetic diversity in *B. attenuata* populations spanning a broad range of precipitation, temperature, and fire regimes (mean fire intervals) in SWA. Our approach detects SNPs that show concordant differences in allele frequencies across populations with respect to specific local climates and fire regimes. We screened those genetic variations that are putatively associated with genes under directional selection and then used environmental association analysis to identify putative genes associated with adaptation to specific precipitation levels, temperatures, and fire intervals. Finally, we annotated those genes for their potential biological function. Our study attempts to answer the critical question of whether those alleles that confer adaptation to local climate factors and fire regime occur globally with varying frequencies or whether they are highly localized in specific populations. Our research quantifies genetic variations associated with adaptation to climate and fire regimes, identifies geographic regions that are predicted to be most sensitive to the disruption of current patterns of local adaptation under climate change, and provides critical insight into the evolutionary potential of further climate change for an iconic species in SWA.

## Results

### SNP profiles and genetic variation in *Banksia attenuata* populations

RAD-seq sequencing coverage averaged 16.0 million reads and 1.53 GB of data per individual for each of the 80 individuals of *B. attenuata* sampled across the nine locations. One sample with 2.1 million reads was discarded due to its low coverage. The average quality score (Q20) was 99.4%, with the lowest being 99.1%, suggesting very high quality for the obtained sequences. The final RAD reference genomes generated from the RAD sequences of twenty individuals using *de novo* assembly contained 241,259 contigs, with N_50_ = 264 and a total length of 63.6 million base pairs. The RAD reference genome had GC contents of 38.0%.

**Figure 1:**
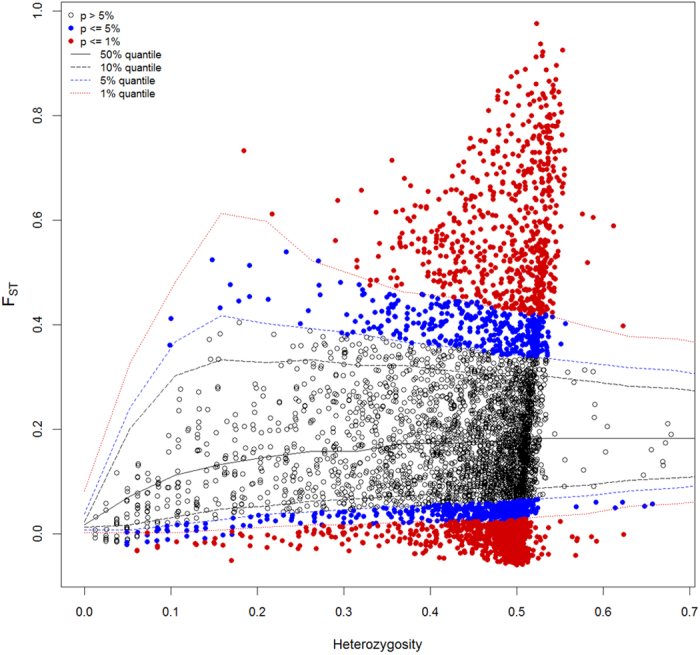
Detection of loci (SNPs) under selection from genome scans using the Fst-outliers approach.

A total of 9887 SNPs passed initial filters in our variant calling approach. We identified 5701 SNPs that existed in all 80 sampled individuals. Overall, 71.9% of the SNPs were biallelic, 27.5% were tri-allelic, and 0.5% contained more than three alleles. Applying a stringency of *P* = 0.01, the F_st_-outlier approach using a hierarchical population model and coalescent simulations identified 560 SNPs (9.8%) putatively under directional selection (Fig. 1).

**Figure 2:**
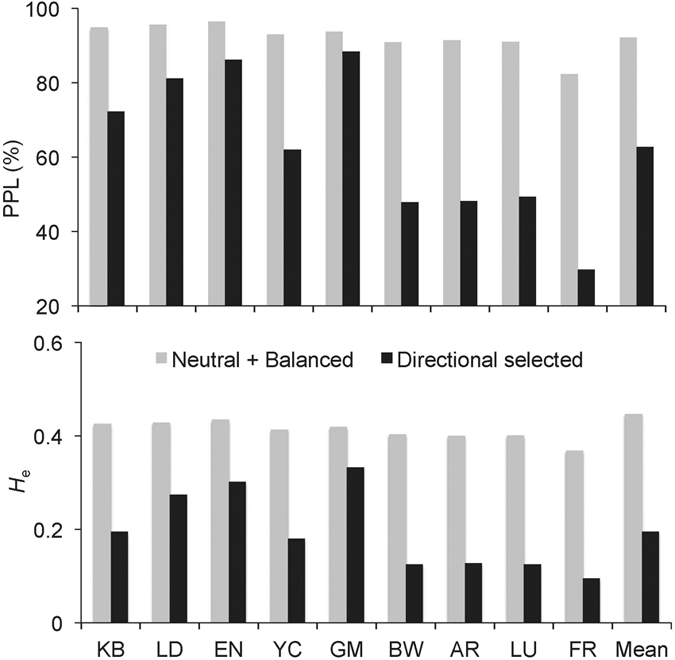
Genetic variation in nine populations of *Banksia attenuata* putatively under balanced or neutral selection, directional selection, as measured by SNPs: A) percentage of polymorphic SNPs; B) expected heterozygosity.

**Figure 3:**
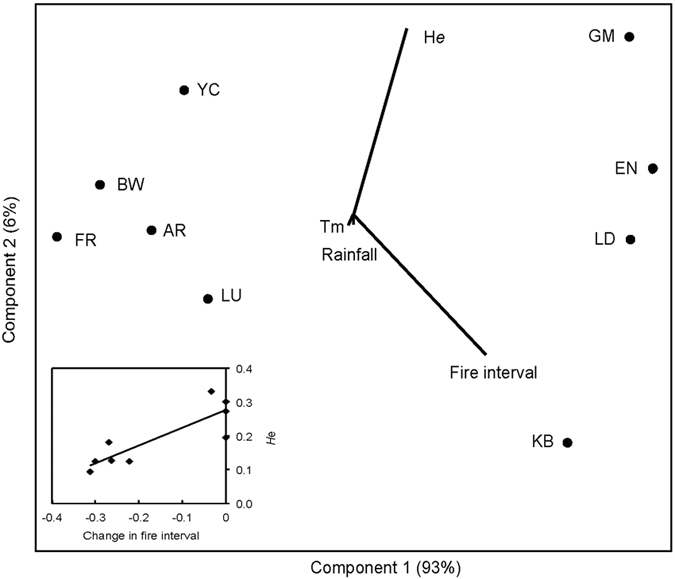
A multidimensional scaling ordination of adaptive genetic variation in nine populations of *Banksia attenuata*, with effects of changes in local climate and fire interval overlaid as correlated environmental vectors.

Adaptive genetic diversity, as measured by the 560 SNPs putatively under directional selection, showed considerable differences among populations. PPL varied from 28% in the population at FR to 81% at GM, with an average of 59%, and expected heterozygosity *H*_e_ ranged from 0.12 at FR to 0.36 at GM and averaged 0.21 (Fig. 2). Population genetic diversity measured by the rest of the SNPs (neutral or possibly under balanced selection) was uniform across all populations other than population FR, which showed lower PPL and *H*_e_ (Fig. 2). Multiple Linear Regression analysis suggested that the level of adaptive genetic diversity (*H*_e_) in each population was largely determined by the change in fire interval (*R*^2^ = 0.734, *P* = 0.003) and not by change in local climate (*R*^2^ = 0.004, *P* = 0.982 for rainfall; *R*^2^ = 0.063, *P* = 0.264 for high temperature) (Fig. 3). The five populations with lower *H*_e_ (YC, BW, SR, LU, FR) have much more frequent contemporary fires compared with historical fires, whereas fire intervals in the other four populations have not changed significantly.

**Figure 4:**
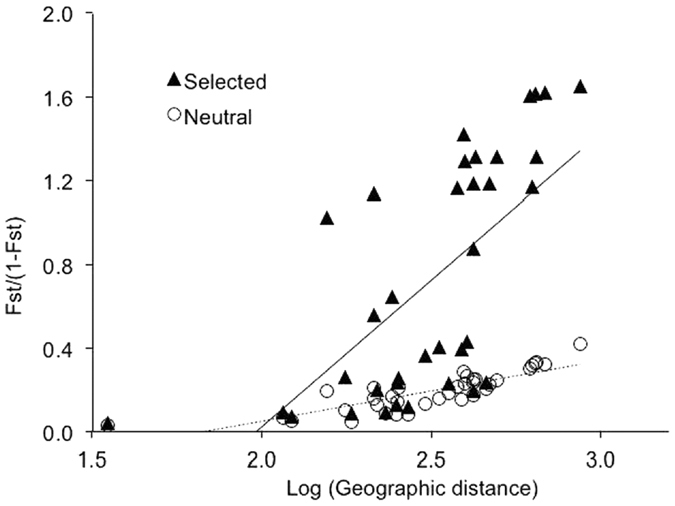
Correlation of pairwise population genetic distance and geographic distance among nine *Banksia attenuata* populations in SW Australia.

For the 5141 neutral loci, population pairwise F_st_ values ranged from 0.003 to 0.342 and averaged 0.113. Populations are significantly differentiated, with all pairwise F_st_ values statistically greater than zero. SNPs putatively under directional selection revealed much greater genetic differentiation among populations, with pairwise F_st_ values ranging from 0.086 to 0.809 and averaging 0.438. For both measures, the geographic distance between populations has contributed to genetic differentiation, with differentiation increasing with increasing geographic distance between populations (Fig. 4).

Environmental correlation using *Bayevn* identified 25 SNPs as significantly associated with rainfall gradients, 18 with maximum temperature, 37 with extreme high temperature, and 11 with historical fire intervals. A total of 14 SNPs are associated with at least two climate factors associated with temperature and solar exposure, whereas only one SNP was associated with rainfall and temperature. SNPs associated with historical fire regime were also specific, with only one of 25 associated with high temperature and one with rainfall.

**Figure 5:**
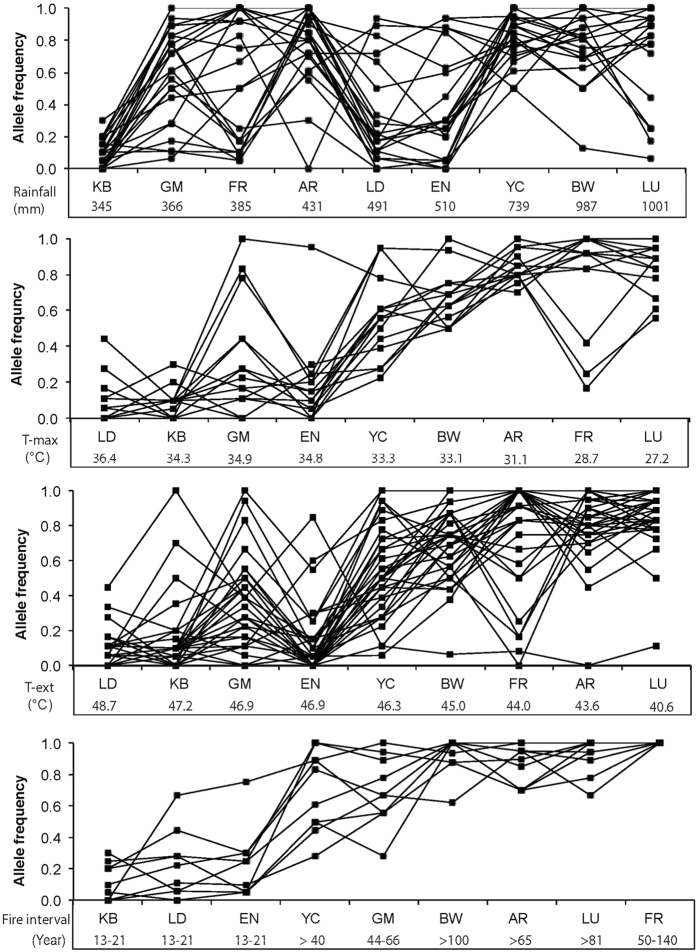
Distribution of allele frequencies for SNPs associated with climate and fire intervals in nine populations of *Banksia attenuata*. Note: only one allele per SNP loci is plotted.

The majority of the SNPs associated with rainfall, maximum temperature, and extreme temperature were not fixed in one allele in a specific population, whereas SNPs associated with specific fire intervals were mainly fixed in one allele (Fig. 5). A total of 20% of SNPs for rainfall and 17% for maximum temperature and extreme temperature together were fixed at one allele in a specific population, whereas overall, 34% of the SNPs that were associated with specific fire intervals were fixed in specific populations. In the population at FR, 10 out of the 11 SNPs were fixed in one allele, with the remaining SNP severely skewed toward one allele (with a frequency of 0.83).

Aligning the results here with the annotated *Banksia hookeriana* leaf transcriptome revealed a total of 16 candidate genes with biological functions, including four candidate genes with functions for adaptation to rainfall, 16 to high temperature and solar exposure, and two to fire intervals (Table 1). Among these, the malate dehydrogenase (MDH) gene was identified as important in adaptation to both rainfall and high temperature. The sphingosine-1-phosphate phosphatase (S1P) gene was linked to adaptation to rainfall, and the 5′ AMP activated protein kinase (AMPK) gene was identified as important in adaptation to high temperature. For fire intervals, the multi-functional guanine nucleotide exchange factor (GEF) gene was one of the most important candidate genes.

## Discussion

### Frequent fire depletes adaptive genetic diversity in *Banksia attenuata*

Our results suggest that altered fire regime, and particularly shortened fire intervals in some parts of SWA, has had a significant impact on the level of adaptive genetic variation in populations of the widespread and abundant *Banksia attenuata*. The five populations in the southern forests of SWA all show a much lower adaptive genetic variation (*H*_*e*_) as measured by 560 SNPs putatively under directional selection than those of populations from the northern part of the species’ geographic range. Principal components analysis suggests that the change in fire interval was the main driver of decline of adaptive genetic variation in *B. attenuata* populations. Recurrent fire has been a major evolutionary force in the evolution of terrestrial plants for at least 100 million years^17^,^47^,^48^. However, plants are not adapted to fire *per se* but, rather, to specific fire regimes that include fire frequency, fire intensity, and patterns of fuel consumption^17^. It has been argued that increasing fire frequency intensifies the selection on plant species in these environments^17^. This effect has likely led to depletion of adaptive genetic variation in some *Banksia attenuata* populations in SWA, where fire occurrence has increased as a result of human influence, but not in others where historical fire regimes remain largely intact.

Multiple lines of evidence support the idea of dramatic changes in fire interval in the southern forests of SWA. Burrows *et al*. reported a mean fire interval of approximately 80 years for tree-scarring fires in jarrah (*Eucalyptus marginata*) forests of SWA in the pre-European period^49^. More recently, the fire interval has decreased due to a combination of more frequent wild fires and regular prescribed burning, which is implemented in this region for a range of land management objectives^21^. Hobbs also posits a change in fire regime from infrequent (~50 years) to frequent (6–8 years) following the European settlement in temperate banksia woodlands of SWA^50^. Aboriginals most likely did not permanently occupy the semi-arid shrublands of Fitzgerald River National Park in the pre-European period due to food resource limitations, and analyses of charcoal from sediment cores indicate intervals between major fires of 50 to 140 years^49^. Similarly, kwongan vegetation in northern SWA is low in resource availability from the perspective of Aboriginals and would not have warranted regular occupation and “management” using fire^19^. Field observations and analysis of satellite imagery over the past 40 years suggest that fire intervals in kwongan vegetation average approximately 13 years^49^.

Palynological evidence suggests that the Proteaceae-Myrtaceae “Kwongan” scrub vegetation (community dominants include *Acacia, Banksia, Casuarina, Eucalyptus, Grevillea, Melaleuca* and *Xylomelum angustifolium*) has changed little since 2.9 Ma in Yallalie, SWA, a location close to the populations at Eneabba (Beekeepers Nature Reserve and South Eneabba Nature Reserve) used in our study. The mid-Pliocene fire interval at Yallalie was proposed to be slightly longer than 10 years^50^, suggesting an incredibly reliable fire return time in this region over evolutionary timescales.

The juvenile period in woody species is generally correlated with longevity, such that longer-lived species have longer juvenile stages^51^. Thus, if the fire interval between successive fires is shorter than the time required for long-lived woody species (recovering from fire either by resprouting or from seed) to mature and set seed, the abundance of these species will decline^24^,^52^. Enright *et al*. estimated that individuals of *Banksia attenuata* near Eneabba, SWA, may live for up to 300 years^53^. Field observations show that the secondary juvenile stage (resprouting of existing individuals after fire) lasts 2–3 years, with little or no viable seed available until at least four years following a fire. Thus, few seedlings will be recruited for fires at 3–5 year intervals. Frequent fires would further impact long-term population size, as seedlings are more vulnerable to fire than are resprouts. Frequent fires can also deplete carbohydrate stores in resprouting species^54^, resulting in reduced survivorship and vigor. With the increasing selective pressure from shortened fire intervals, only individuals with beneficial alleles survive, leading to a *de facto* selective sweep.

Genome-wide analysis of adaptive genetic variation in *Banksia attenuata* revealed a clear signature of increased fire frequency as a consequence of fire management, first by Aboriginal people, then European settlers, to the current government fire management agency programs. We identify the presence of higher adaptive genetic variation in populations where fire frequency has been relatively stable over long periods of time but much lower genetic variation in populations where fire has become more frequent as a result of changes to the mean fire interval. Because changes in fire regime in certain parts of SWA have been relatively recent, new adaptive mutations are not likely to have appeared, leaving the species reliant on existing genetic diversity to facilitate persistence. High adaptive genetic variation must have existed in those populations (as it continues to do in some others reported here). This contemporary evolutionary response to frequent fire has reduced variability at the selected loci. Such selective sweeps may reduce the population’s ability to respond genetically to future fluctuations in fire regime, leading to unpredictable effects on the species’ presence and abundance at the level of the plant community. Given the current very low level of adaptive genetic variation in populations in southern SWA, the capacity to survive more frequent fires is highly uncertain.

### Adaptive variation to rainfall and high temperature

Rapid environmental changes have long been recognized as powerful driving forces for positive directional selection^55^. Directional selection is reliant on the availability of genetic diversity upon which selection can act. Our genome-wide study has revealed high levels of adaptive genetic variation in populations of *Banksia attenuata* across its range. Most of the candidate genes (~80%) associated with rainfall and high temperature have multiple alleles, and the results of our principal components analysis suggest that recent changes in rainfall and temperature have had little impact so far on within-population adaptive genetic variation. These results are perhaps not surprising because inter-annual fluctuations in rainfall and temperature are normal occurrences and different genotypes of a species may be favored in different years. Inter-annual environmental fluctuations may be one driver by which functional genetic variation is maintained in natural populations^56^. The regions inhabited by *B. attenuata* range from semi-arid with an annual rainfall of 360 mm to the high rainfall zone with over 1000 mm annually, supporting the interpretation that species spanning wide climate range may have greater intrinsic adaptability due to high adaptive genetic variation. In *Arabidopsis thaliana*, Lee & Mitchell-Olds similarly demonstrated that environmental adaptation contributes to gene polymorphism across the genome^14^. Given the existence of such diverse climate-related genetic variability within natural populations of *B. attenuata*, its capacity to adapt to changes in climate (declines in rainfall and rising temperatures) may be large.

The impacts of declining rainfall on plant species may be greater in combination with increasing temperatures, prompting the hypothesis that genes that confer fitness under drought stress may overlap with those associated with tolerance to high temperatures. However, our results show that the opposite is true for *B. attenuata*. Although there is considerable overlap of the SNPs associated with the mean temperature of the hottest month, with 14 SNPs (out of 45) associated with at least two of these climate factors, of the 25 SNPs associated with annual rainfall, only one was associated with temperature. Such weak genetic correlations may allow traits to respond to selection independently^57^.

Over the last four decades, an unplanned experiment has shown the impact that increasing temperatures, declining rainfall and retreating groundwater levels may have had on *Banksia* species in woodlands near Perth, SWA. Extensive deaths of mature individuals, including *B. attenuata*, have been recorded since the 1970s^58^. However, no *Banksia* species have become locally extinct during the more than 40 years of continuous decline in the water table. Furthermore, new individuals that became established under the changed ecohydrological state of lower groundwater availability have been less stressed by drought compared with their parent populations^59^. In *Eucalyptus*, experimental trials indicate that climatic tolerances of the species may be greater than suggested by their natural distributions^11^. For example, Booth *et al*. showed that *Eucalyptus regnans* was able to grow well at trial sites where the annual mean temperature was 5 °C warmer than the hottest location in its natural distribution^60^. Taken together, our results showing a high level of adaptive genetic variation and an abundance of alleles in those candidate genes associated with adaptation to rainfall and high temperature suggest an intrinsic adaptability in populations of *B. attenuata* to tolerate further changes in rainfall and temperature.

### Ecologically important genes in adaptation to climate and fire regimes

Among the candidate genes that have been identified with close associations to both climate (rainfall and high temperature) and fire intervals in *B. attenuata*, four candidate genes have clear implications for molecular functions in the adaptation to stress as revealed by experimental studies in many other plants. The malate dehydrogenase (MDH) gene was identified as closely related to rainfall variation and high temperature in our study. Previous studies have shown that MDH is sensitive to abiotic stresses and that the expression of MDH is positively correlated with the growth vigor of plants and cells under stress^61^,^62^. It is likely that the enrichment of the MHD gene in *B. attenuata* populations confers a substantial capacity for the species to adapt to a broad spectrum of rainfall and high temperatures, including adaptation to dry and hot environments such as in the northern sandplain of SWA. The sphingosine 1-phosphate phosphatase (S1P) gene has been identified as specifically linked to adaptation to rainfall variation in *B. attenuata*. The S1P gene has important functions in controlling stomatal opening and closure^63^,^64^. Stomatal closure has negative effects on CO_2_ uptake, photosynthesis, and transpirational cooling as well as on water and nutrient uptake. The ability to close the stomata during unfavorable conditions (usually drought stress) represents an important intrinsic adaptation to repeated drought in *B. attenuata.*

High temperature causes a negative carbon balance, which increases the risk of carbon starvation^65^. It is not surprising that 5′ AMP-activated protein kinase (AMPK) has been identified as closely associated with adaptation to high temperature (the temperature in the hottest month) in *B. attenuata*. High temperatures promote stomatal closure, which leads to decreased CO_2_ uptake and subsequently lowers net photosynthesis. AMPK is a sensor of energy status and switches on catabolic pathways that generate ATP, which maintains cell survival during energy starvation^66^. The guanine nucleotide-exchange factor gene (GEF) has been found to be important in morphogenesis, including the regulation of root growth, lateral root formation, root hair differentiation and floral organ formation, and regulation of the formation of plant vascular networks^67^. The GEF gene has been identified as closely associated with fire interval, reflecting its critical role in promoting and regulating post-fire growth and survival in *B. attenuata*.

Eckert *et al*. investigated the genetic basis of climatic adaptation in loblolly pine (*Pinus taeda*) by evaluating the associations between environmental clines and allelic variation using genome-wide markers; they revealed five loci that were significantly associated with aridity gradients^68^. These genes were putatively orthologous to the *Arabidopsis (Arabidopsis thaliana*) genes that confer stress tolerance. Our genome-wide scans identified candidate genes that are related to stress tolerance, the regulation of stomatal closure, energy use, and morphogenesis in the adaptation to climate and fire regime in *B. attenuata*. Further research points to experimentally investigating and validating the functional and physiological pathways of these candidate genes.

### Methodological considerations

The present effort of searching candidate genes involved in the adaptation to climate and fire regimes in SWA must be considered as preliminary, and the list of candidate genes is far from complete. Although our RAD-seq approach has generated data equivalent to a 2–3× coverage of the genome, the reference genome obtained *de novo* from RAD sequences was only 65 MB, equivalent to approximately one-tenth of the whole genome (assuming a genome size of 650–850 MB for *Banksia*). The resulting 5701 SNPs scattered across the *g*enome may be sufficient for providing an overall evaluation of the level of adaptive genetic variation within populations of *B. attenuata* but may not be sufficient to cover all the genes that confer adaptive fitness to climate and fire regime. Furthermore, to facilitate the comparison of the genetic variation among populations and across the range of *B. attenuata*, only those SNPs that were present in all sampled individuals and populations were investigated, which may omit population- or individual-specific genetic variations. Indeed, over 61,000 SNPs (61,000–74,000) were discovered in individual samples, as estimated from the raw RAD sequences. Future advances in analytic methodology may make full use of all the data generated from high-throughput sequencing. Finally, many of the candidate genes in this study are still not well characterized at the functional or transcriptional level. Indeed, among the 18 candidate genes identified by aligning to the *Banksia hookeriana* leaf transcriptome, only the function for four genes can be connected to adaptation to local climate and fire regime. Future research on a fully annotated *Banksia* genome is anticipated to provide a critical platform for the ecology, evolution and adaptation of this iconic genus.

## Conclusion

Genetic variability has the capacity to buffer species against specific environmental changes. Our study was to detect a high level of adaptive genetic variation and candidate genes with a clear ecological function associated with adaptation to local climate and fire regimes in natural plant populations. Our results suggest that *B. attenuata*, and most likely other species with a similar life history and distribution, may be able to tolerate further changes in rainfall and temperature based on adaptive genetic variation within populations. This is corroborated with the results from studies of the impacts of declining water availability on banksias^58^,^59^ and paleo-evidence of climate change and the persistence of banksias *in situ* for almost 3 million years^30^. Our results contribute to the recently proposed notion that some species and ecosystems might be more resilient to climate change than we currently believe, with genetic adaptation leading to “effect dampening” within a relatively short time frame^69^.

Our results reveal that shortened fire intervals, predominantly a consequence of recent human activities, imposed the strongest selection pressure on *B. attenuata* populations in southern SWA. Frequent fires have been driving changes in gene frequency within natural plant populations and have led to selective sweep. Given the current very low level of adaptive genetic variation in those populations of southern SWA, the capacity to survive more frequent fires and further environmental fluctuations in their habitat is substantially reduced. Even if adaptive genetic variations exist in *B. attenuata* populations and an evolutionary response to further climatic changes can occur, this may not be sufficient to ensure the survival of the population. The projected decline in rainfall in SWA, in conjunction with the continuing rise of summer temperature, may result in longer fire seasons and increased fire likelihood, thus further shortening fire intervals^21^.

Finally, despite the presence of positive correlations in heterozygosity as measured by neutral SNPs and by SNPs putatively under directional selection, the pattern of neutral genetic variation in populations of *B. attenuata* was not representative of adaptive genetic variation, particularly in those populations experiencing shortened fire intervals in the southern part of SWA. Our results highlight the long-held concerns regarding the use of neutral genetic variation as a surrogate for adaptive genetic variation.

## Materials and Methods

### Species and sampling

**Figure 6:**
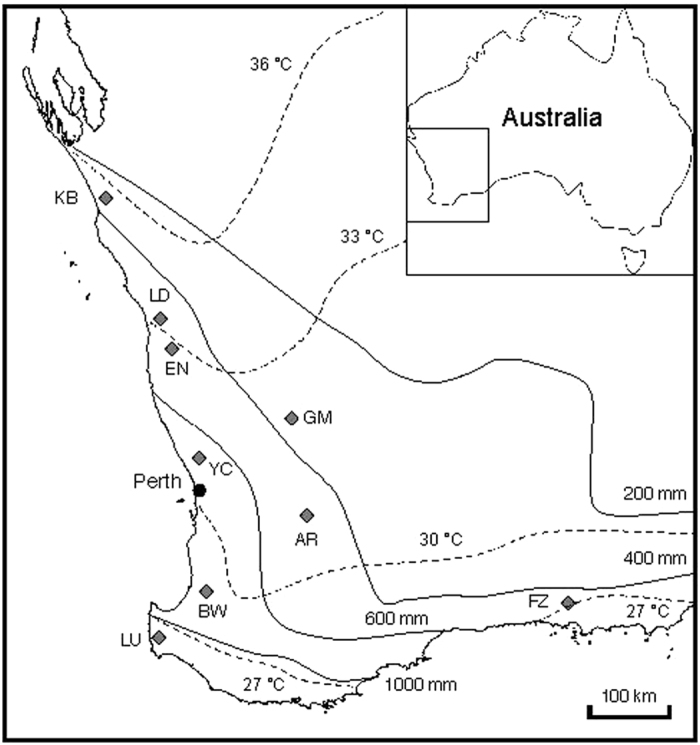
Locations of the sampled populations of *Banksia attenuata* in Western Australia. Continuous lines indicate annual rainfall isohyets, and broken lines indicate isotherms of average temperatures for February. Annual rainfall and temperature data represent a 30-year average (1980–2010) and are from the Australian Bureau of Meteorology. Map was created using Adobe Illustrator CC based on outline map available from The University of Melbourne Library Map Collection (http://www.lib.unimelb.edu.au/collections/maps/digital/outline-maps/).

*Banksia attenuata* (Proteaceae) is a member of the iconic Australian genus *Banksia*, an important element of the flora, with over 180 species in SWA. This species forms an important component of open *Eucalyptus* and *Banksia* woodlands and shrublands as a dominant or understory tree or tall shrub. The distribution of *B. attenuata* spans a wide climate and environmental range, and it is the most widely distributed of all western banksias. *B. attenuata* is found across much of SWA west of the 400 mm isohyet, with a few populations penetrating slightly east into areas with less than 400 mm annual rainfall, through to the west coast of WA, north to Kalbarri National Park (with an annual rainfall less than 400 mm), south to Cape Leeuwin (>1000 m annual rainfall) and across to the Fitzgerald River region (Fig. 6). This species has an evolutionary history of ~19 million years and is one of the oldest members of the extant *Banksia*^47^, implying a historically strong capacity to adapt to climatic and environmental changes. Individuals of *B. attenuata* are estimated to live for 300 years or more^53^ and can disperse seeds up to at least 2.6 km (1.6 miles) in a single dispersal event^25^.

**Figure 7:**
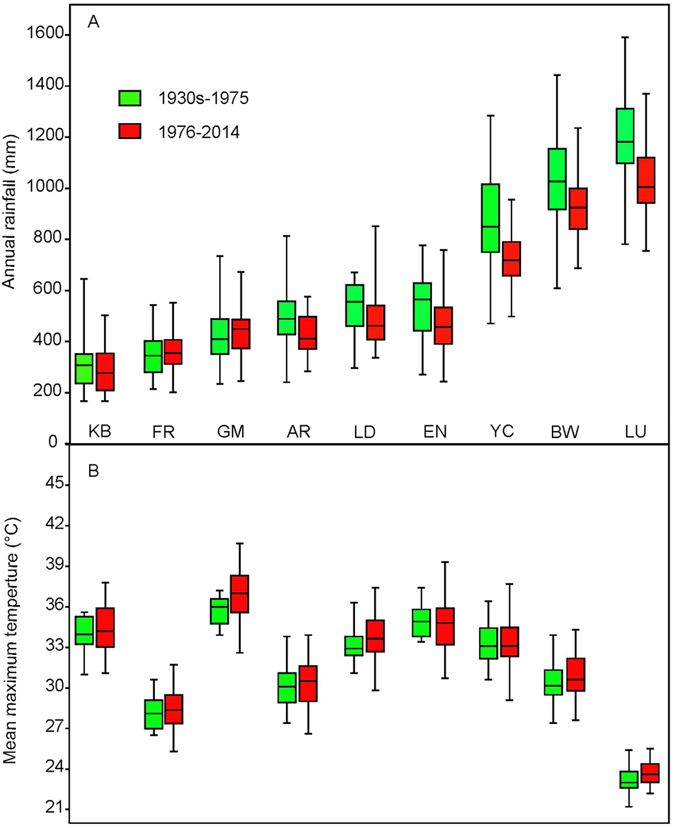
Change in annual rainfall (**a**) and mean maximum summer temperature (**b**) since 1975 at nine sampling locations. Data are from the Australian Bureau of Meteorology.

**Figure 8:**
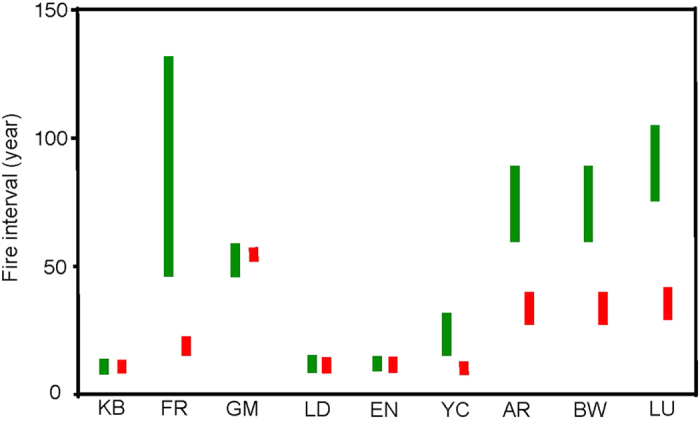
Changes in fire regime at nine sampling locations. Green bars indicate variations in historical fire intervals; red bars indicate the contemporary interval. Data was complied from refs 18, 19, 70, 71, 72, 73.

Up to ten individuals of *Banksia attenuata* were randomly sampled from each of nine locations across its range in SWA (Table 2; Fig. 6). These nine locations span a rainfall gradient from 330 mm to over 1000 mm and cover three major vegetation types (shrubland, woodland, and forest). Long-term weather data recorded at the nearest weather stations were obtained from the Bureau of Meteorology of Australia (www.bom.gov.au). Long-term weather observations (since the 1930s) show a marked decline (15–30% reduction) in annual rainfall since 1975 in seven of the nine locations (Fig. 7a), and a 0.5–1 °C increase in mean temperature in the hottest month (February) in all nine locations compared with pre-1975 (Fig. 7b). Historical fire intervals range from ~10 years in the north to 140 years in the south. Data for contemporary fire intervals indicate major changes in the southern (shortened fire intervals) but little change in the northern parts of the species’ geographic range (Fig. 8; data for fire intervals from^18^,^19^,^70^,^71^,^72^,^73^).

### RAD-seq and SNP discovery

Genomic DNA from each individual was extracted and then fragmented by the corresponding enzyme (E*coR*1, recognition site: 5′-G/AATTC-3′). EcoRI is a frequent cutter, resulting in the detection of more markers in RAD sequencing. For library construction, two 100 bp single-end sequencing libraries were constructed using the eight-nucleotide multiplex identifiers. Each library contained five individual samples. Each sample was assigned to a unique MID barcode. The RAD products from the 80 plants were processed on an Illumina HiSeq2000 platform (Illumina Inc., San Diego, CA, USA) at Beijing Genomics Institute (Shenzhen, China). Sequencing data were segregated by individual specific MID. Reads from each plant were clustered into tag reads by sequence similarity (allowing two mismatches at most between any two reads within each tag reads cluster). To ensure quality, the raw data were modified by the following two steps: first, the adapter pollutions and index sequence in the reads were deleted, and then the reads which contained more than 50% low-quality bases (quality value ≤ 5) were discarded.

Because there is no reference genome available for banksias or closely related taxa, a *de novo* RAD reference genome was constructed. Reitzel *et al*. demonstrated that the results from analyses with and without a reference genome detect similar sets of SNPs^46^, highlighting that RAD-seq can be efficiently applied to species lacking existing genomic resources. RAD-seq reads from 20 randomly chosen samples were used for *de novo* assembly. At the initial step, pair-reads were collapsed into RAD sequence clusters if the SE (first rad tag) shared 100% sequence identity across the Illumina reads. The RAD sequence cluster was set at a range of 50–750× to maximize the efficient assembly of sequences. The paired-end sequences corresponding to the selected SE were extracted for further assembly. The selected pair-end reads were input into the Velvet sequence assembler, and k-mer 40 was used for *B. attenuata* contig assembly^72^. Assembled contigs less than 200 bp were excluded from further analysis.

The final filtered RAD reference genome assemblies should thus represent single-copy genomic sequences. Reads for each individual were then aligned to the RAD reference using BOWTIE^74^,^75^, again using sequence quality information and allowing a two mismatch maximum as well as permitting alignment to no more than one reference region per read. SAM tools were used to convert Bowtie alignments into BAM and pileup files for SNP identification^76^. Sequence variants from the pileups were then condensed into a variant call format (VCF) file. For an SNP to be recognized, it had to appear in all sequenced samples.

### Analysis of adaptive genetic variation

To assess the presence and extent of adaptive genetic variation in populations, we began by asking how many SNP loci have diverged under selection in *B. attenuata*. A locus under balancing selection should show uniform allele frequencies across populations, whereas loci under local directional selection should show large differences among populations. We used the F_st_-outlier approach to detect SNP loci that are putatively under selection, and these markers were expected to reveal a signal of adaptive variation related to local climate and environment^77^,^78^. F_st_-outlier identification followed the approach of hierarchy modeling and coalescent simulation^77^ and was implemented in Arlequin v3.5^79^,^80^. Briefly, coalescent simulations were used to obtain a null distribution and confidence intervals around the observed values and to determine whether observed locus-specific F_st_ values could be considered as F_st_ outliers conditioned on the globally observed F_st_ value. The populations in our samples were separated by large geographic distances and could be considered as independent units, which minimizes false positives in hierarchy modeling and coalescent simulation^80^. Using a stringency of *P* = 0.01, we categorized SNPs into three groups: SNPs under directional selection, those under balanced selection, and neutral SNPs. For each subset of SNPs, population genetic variation (percentage of polymorphic loci, PPL; expected heterozygosity *H*_e_) and pairwise population differentiation (F_st_) were estimated in Arlequin v3.5^80^. To test isolation by distance, a correlation analysis was performed between pairwise population differentiation F_st_, as estimated from SNPs that were detected under directional selection, F_st_ estimated from those SNPs other than under directional selection, and geographical distance (transformed using the logarithm function).

To investigate whether climate change (i.e., declining rainfall and rising temperature) since ~1970 and changes in fire interval have impacted the level of adaptive genetic diversity in each population, we used multiple linear regression with adaptive genetic diversity (expected heterozygosity, He) as dependent variable, and change in climate (annual rainfall and average temperature in the hottest month, usually February) and fire interval as independent variables.. Climate change was quantified as [Mean (post-1975) − Mean (pre-1975)]/[Mean (post-1975) + Mean (pre-1975)]. Change in fire interval was based on the historical versus contemporary intervals (Table 1) and used the same formula. Multiple Linear Regression was performed in PAST^81^, with a bonferroni correction employed for multiple comparisons. A multidimensional scaling ordination, drawn using PAST^81^, was used to illustrate associations among variables.

The subset of SNPs putatively under directional selection was further used to detect specific candidate genes associated with adaptation to annual precipitation, temperature (mean maximum temperature of hottest month, and hottest temperature), solar exposure, and historical fire intervals using environmental associations^82^,^83^. Association with contemporary fire intervals was not examined because *B. attenuata* is a long-lived plant with a long generation time (the sampled individuals were adult trees and likely more than 100 years old). We employed a Bayesian method that estimates the empirical pattern of covariance in allele frequencies between populations from a set of markers and then uses this as a null model for a test of individual SNPs^83^. The Bayesian method uses environmental correlations to identify underlying local adaptation of loci and largely overcomes problems of differences in sample sizes and the neutral correlation of allele frequencies across populations due to shared history and gene flow^83^. Both simulation and empirical datasets suggest this approach is very useful for identifying selected loci via their correlation with environmental variables and can be applied to continuous or discrete environmental variables^83^. Analysis of environmental association was implemented in *Bayenv* (http://gcbias.org/bayenv/). Only one SNP locus from the same contig was used for environmental association.

### Annotation of genes putatively associated with rainfall and fire regime

We further investigated the current functional annotation and classification of the candidate genes for local adaptation as revealed in the above environmental associations. The *de novo* assembled contigs of *B. attenuata* were used for BLASTN searches and annotation against an annotated *B. hookeriana* transcriptome assembly (obtained from http://www.ncbi.nlm.nih.gov) using an E-value cut-off of 10^−10^ (E-value < 10^−10^)^84^. *B. hookeriana*-annotated transcripts’ information (Nr protein database similarity, GO annotation and KEGG pathway annotations) was applied to *B. attenuata* contigs if they were matched against each other.

## Additional Information

**How to cite this article**: He, T. *et al*. Evolutionary potential and adaptation of *Banksia attenuata* (Proteaceae) to climate and fire regime in southwestern Australia, a global biodiversity hotspot. *Sci. Rep.* **6**, 26315; doi: 10.1038/srep26315 (2016).

## Figures and Tables

**Table 1:** SNPs and corresponding candidate genes for *Banksia attenuata* from 9 populations in SWA that have been annotated and whose molecular function and biological processes have been identified.

A. Related to rainfall					
**Name**	**Bayes Factor**		**KEGG-related Pathway**	**Biological Process**	**Molecular Function**
SNP066	3.6				ATP-dependent DNA helicase HFM1/MER3 [EC:3.6.4.12]
SNP086	3.7			Methionine biosynthetic process Proteolysis RNA splicing	Lysosomal ProX carboxypeptidase [EC:3.4.16.2] Carboxypeptidase activity Erine-type peptidase activity
SNP120	7.8		Metabolic pathways Biosynthesis of secondary metabolites Pyruvate metabolism Carbon fixation in photosynthetic organisms Glyoxylate and dicarboxylate metabolism Citrate cycle (TCA cycle)	Response to salt stress Defense response to bacterium Response to cold Malate metabolic process Tricarboxylic acid cycle Cellular carbohydrate metabolic process Response to cadmium ion	Malate dehydrogenase [EC:1.1.1.37] Copper ion binding L-malate dehydrogenase activity Nucleotide binding
SNP246	3.0			Sphingolipid metabolic process Stomatal closure Response to abscisic acid stimulus	Sphingosine 1-phosphate phosphatase activity
**B. Related to adaptation to temperature**					
**Name**	**T-max**	**T-ext**	**KEGG-related Pathway**	**Biological Process**	**Molecular Function**
	**Bayes Factor**				
SNP036	3.0	3.1			Proteasome component ECM29
SNP108	1.9	3.8	RNA transport	Negative regulation of biological process Protein import into nucleus Organ morphogenesis; Xylem and phloem pattern formation Flower morphogenesis Determination of bilateral symmetry Protein targeting to chloroplast	Nuclear pore complex protein Nup62 Transmembrane signaling receptor activity Hydrolase activity Acting on acid anhydrides
SNP120	0.8	3.1	Metabolic pathways Biosynthesis of secondary metabolites Pyruvate metabolism Carbon fixation Glyoxylate and dicarboxylate metabolism Citrate cycle (TCA cycle)	Response to salt stress; Defense response to bacterium; Response to cold; Malate metabolic process; Tricarboxylic acid cycle; Cellular carbohydrate metabolic process Response to cadmium ion	Malate dehydrogenase [EC:1.1.1.37] Copper ion binding; L-malate dehydrogenase activity Nucleotide binding
SNP214	1.3	4.3		Protein ubiquitination Protein phosphorylation	Interleukin 1 receptor-associated kinase 4 [EC:2.7.11.1]; ATP binding, ubiquitin protein ligase activity; Protein serine/threonine kinase activity
SNP230	2.8	3.1			Structure specific endonuclease subunit SLX1 [EC:3.6.1.]
SNP238	1.0	8.4			tRNA (cytosine 38C5) methyltransferase [EC:2.1.1.204]
SNP303	5.2	1.7		Cellular response to glucose starvation; Protein autophosphorylation	5′AMP activated protein kinase, Regulatory gamma subunit; Protein binding; Protein kinase activator activity; Protein serine/threonine kinase activity
SNP306	0.7	5.4		Borate transmembrane transport Response to boron-containing substance	Inorganic anion exchanger activity; Borate efflux transmembrane transporter activity
SNP311	5.3	12.0	Metabolic pathways Pyrimidine metabolism	DNA dependent transcription Nucleotide phosphorylation	UMPCMP kinase [EC:2.7.4. 2.7.4.14] ATP binding, Nucleoside triphosphate adenylate kinase activity; Nucleotide kinase activity
SNP348	3.2	6.3		Embryo development ending in seed dormancy Microtubule cytoskeleton organization	Transferase activity
SNP355	3.7	1.0	Metabolic pathways Biosynthesis of secondary metabolites Stilbenoid Diarylheptanoid and gingerol biosynthesis Limonene and pinene degradation		
SNP412	2.3	7.7	Circadian rhythm plant	Blue light signaling pathway Proteasomal protein catabolic process Response to red light Regulation of transcription, Flower development	Clock associated PAS protein ZTL Blue light photoreceptor activity
**C. Related to (historical) fire interval**					
**Name**	**Bayes Factor**		**Function-Description**	**Biological Process**	**Molecular Function**
SNP004	4.81		SEC7 domain proteins	Seed maturation Endosome transport via multi-vesicular body sorting pathway Actin nucleation; Lateral root formation; Regulation of chromosome organization Proteasomal protein catabolic process; Regulation of vesicle targeting, to, from or within Golgi Floral organ formation Basipetal auxin transport; Unidimensional cell growth Establishment of planar polarity Regionalization; Trichome morphogenesis Cellulose biosynthetic process Regulation of ARF protein signal transduction; Root hair cell differentiation; Regulation of catalytic activity Vegetative to reproductive phase transition of meristem Regulation of cell differentiation Phloem or xylem histogenesis Longitudinal axis specification Primary shoot apical meristem specification Cell wall organization	Guanine nucleotide-exchange factor GTP: GDP antiporter activity; ARF guanyl-nucleotide exchange factor activity Protein homodimerization activity
SNP361	3.06		Cytochrome P450	Oxidation-reduction process	Heme binding Iron ion binding Electron carrier activity Aromatase activity

**Table 2:** Locations and long-term climate of the sampled *B. attenuata* populations.

**Location**	**Habitat**	**Annual Rainfall (mm)**	**Fire interval (years)**		**T-max**^**a**^ **°C**	**T-ext**^**b**^ **°C**
			**Historical**	**Contemporary**		
Kalbarri (KB)	shrubland	345	15	15–22	34.3	47.2
Leda (LD)	shrubland	491	13–21	13–21	36.4	48.7
Eneabba (EN)	shrubland	510	13–21	13–21	34.8	46.9
Yanchep (YC)	woodland	739	40	12	33.3	46.3
Goomalling (GM)	woodland	366	63	44–66	34.9	46.9
Brunswick (BW)	forest	987	>100	15–20	33.1	43.6
Arthur River (AR)	forest	431	>65	15–20	31.1	44.0
Cape Leeuwin (LU)	forest	1001	>81	15–20	27.2	40.6
Fitzgerald River (FR)	shrubland	385	50–140	22	28.7	45.0

a, mean maximum temperature of the hottest month (February); b, hottest temperature recorded.
